# Preparation of electrochemically active silicon nanotubes in highly ordered arrays

**DOI:** 10.3762/bjnano.4.73

**Published:** 2013-10-16

**Authors:** Tobias Grünzel, Young Joo Lee, Karsten Kuepper, Julien Bachmann

**Affiliations:** 1 Physics Department and Chemistry Department, University of Hamburg, Sedanstrasse 19, 20146 Hamburg, Germany; 2 Physics Department, University of Osnabrück, Barbarastrasse 7, 49076 Osnabrück, Germany; 3 Department of Chemistry and Pharmacy, Friedrich Alexander University Erlangen-Nürnberg, Egerlandstrasse 1, 91058 Erlangen, Germany

**Keywords:** atomic layer deposition, electrochemistry, lithium ion battery electrode, silica thermal reduction, silicon nanotubes

## Abstract

Silicon as the negative electrode material of lithium ion batteries has a very large capacity, the exploitation of which is impeded by the volume changes taking place upon electrochemical cycling. A Si electrode displaying a controlled porosity could circumvent the difficulty. In this perspective, we present a preparative method that yields ordered arrays of electrochemically competent silicon nanotubes. The method is based on the atomic layer deposition of silicon dioxide onto the pore walls of an anodic alumina template, followed by a thermal reduction with lithium vapor. This thermal reduction is quantitative, homogeneous over macroscopic samples, and it yields amorphous silicon and lithium oxide, at the exclusion of any lithium silicides. The reaction is characterized by spectroscopic ellipsometry for thin silica films, and by nuclear magnetic resonance and X-ray photoelectron spectroscopy for nanoporous samples. After removal of the lithium oxide byproduct, the silicon nanotubes can be contacted electrically. In a lithium ion electrolyte, they then display the electrochemical waves also observed for other bulk or nanostructured silicon systems. The method established here paves the way for systematic investigations of how the electrochemical properties (capacity, charge/discharge rates, cyclability) of nanoporous silicon negative lithium ion battery electrode materials depend on the geometry.

## Introduction

A significant research and development effort has been dedicated to the positive electrode materials of lithium ion batteries [1]. In contrast, the negative electrode of all commercial lithium ion batteries still consists of graphite, which can intercalate lithium up to a theoretical stoichiometry LiC_6_ [2]. Silicon, however, can react with lithium to create several phases with stoichiometries as high as Li_4.4_Si [3]. This corresponds to a theoretical lithium storage capacity of 4200 mAh g^–1^, more than 10 times as high as in the case of LiC_6_. Even the number 3000 mAh g^–1^, which is also often mentioned in the literature, is still eight times as high as for graphite [4]. Unfortunately, the significant volume changes that occur upon loading of Si with Li, and which are associated with the concomitant phase transitions, severely constraint the practical exploitation of this very large capacity [5]. In bulk silicon, one does not limit oneself to charging and discharging a small fraction of the theoretically available lithium, the mechanical tensions generated by the full electrochemical cycling will rupture the solid and large fractions of the material will lose the electrical contact.

In principle, this difficulty could be circumvented by nanostructuring. A porous structure in which parallel cylindrical channels run ‘vertically’ from the electrolyte to the vicinity of the current collector should allow for a ‘lateral’ expansion of the electrode material upon charging, whereas direct ‘vertical’ transport paths are maintained for the charge carriers in the solid electrode (for the electrons) and in the electrolyte (for the Li^+^ ions). The transport of Li^+^ ions inside the solid remains ‘horizontal’, so that the lateral characteristic length of the porous structure should be small. Indeed, a proof of principle has been provided based on nanowires and nanotubes obtained either by vapor-liquid solid methods or from bulk silicon [6–8], and based on porous silicon [7,9]. However, no study is available to date in which the geometric parameters of this system were varied systematically in order to pinpoint the critical length scales associated with mass transport, charge transport, and mechanical relaxation.

**Figure 1:**
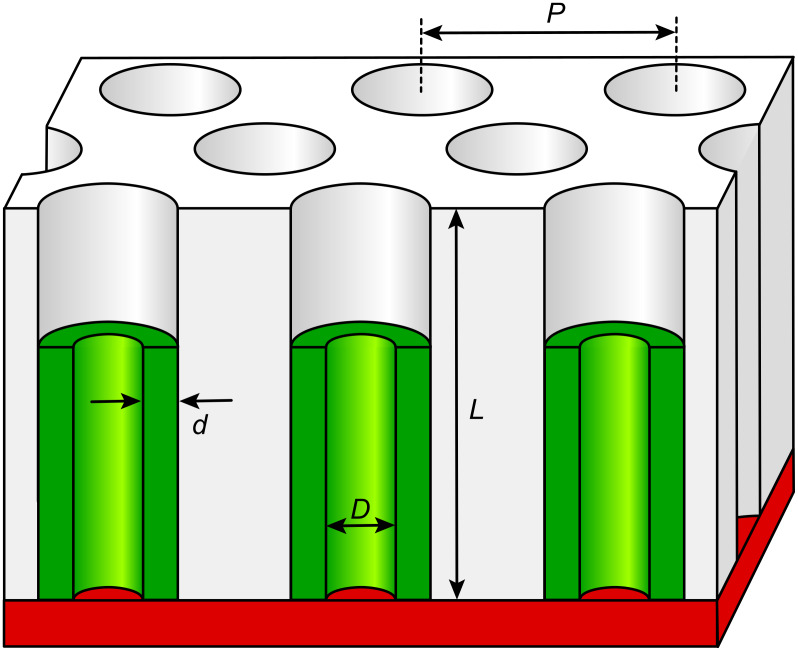
Schematic view of the proposed system, including all tunable geometric parameters. An inorganic matrix (white) defines cylindrical pores of length *L* and in a hexagonal order of period *P*. The silicon tubes (green) have a wall thickness *d* and an inner diameter *D*. The electrical contact is represented in red color.

We propose an experimental platform specifically designed to provide the experimental capability of tuning individually every single geometric parameter in such a porous silicon structure created in an inert matrix (Figure 1): the pore length *L*, the pore diameter *D*, the silicon layer thickness *d*, and the interpore distance *P*. The matrix (white) will be prepared by the two-step anodization of aluminum, a procedure which enables the experimentalist to generate templates of ordered cylindrical pores with a tunable period 50 nm ≤ *P* ≤ 450 nm and a length 0.1 µm ≤ *L* ≤ 100 µm [10–11]. Subsequently, the functional material will be deposited into the pores conformally by atomic layer deposition (ALD). This method based on well-defined, self-limiting surface reactions carried out in a cyclic manner enables one to create films of accurately tunable thickness *d* on the surfaces of such porous substrates [12–15], Because silicon is one of the very few simple inorganic solids for which no practical ALD reaction scheme is available [16], we will deposit SiO_2_ instead [17–18], and then reduce it to elemental silicon. This paper reports on the reduction reaction that we developed based on lithium vapor, which exhibits the following crucial properties: (1) quantitative and homogeneous conversion of the sample, (2) conservation of the nanoscale morphology, (3) facile removal of the byproduct, (4) possibility to be carried out at reasonably low temperature and within a short time.

## Results and Discussion

### Overview of the preparation

**Figure 2:**
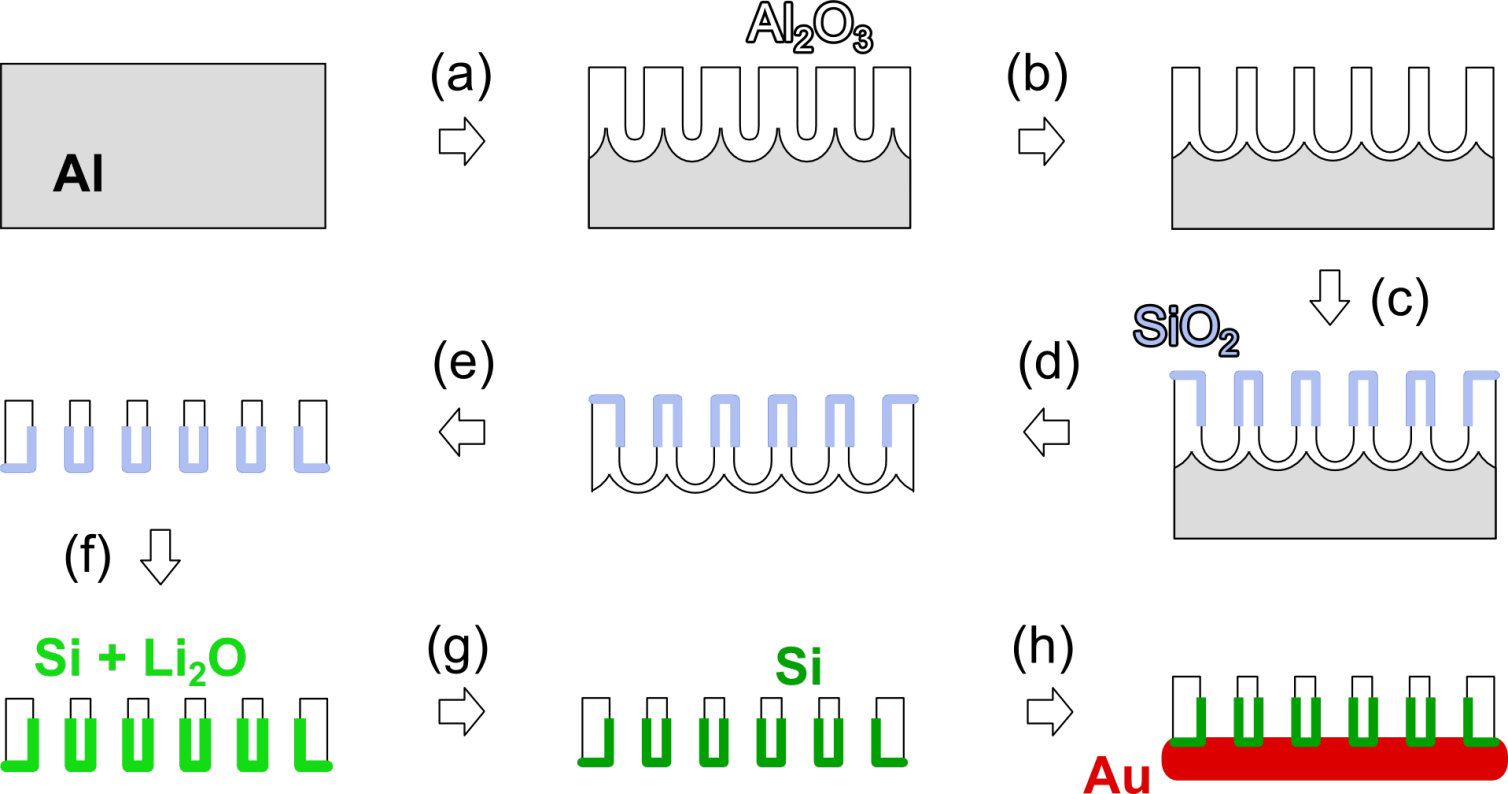
Preparative scheme: (a) –*e*^–^, H_2_C_2_O_4_/H_2_O, 7 °C (two-step anodization); (b) H_3_PO_4_/H_2_O, 45 °C (pore widening); (c) H_2_N(CH_2_)_3_Si(OEt)_3_, H_2_O, O_3_/O_2_, 150 °C (ALD); (d) CuCl_2_/HCl/H_2_O, 20 °C; (e) H_3_PO_4_/H_2_O, 45 °C; (f) Li, 670 °C; (g) HCl/H_2_O, 20 °C; (h) Au sputter, 20 °C. Note that despite the impression which may emanate from this cross-section representation, the aluminum oxide framework remains reticulated and continuous throughout (see Figure 1).

The preparative path devised for making ordered arrays of electrically contacted silicon nanotubes is presented in Figure 2. In the first step (a), a double anodization (electrochemical oxidation of aluminum in a protic solution) is carried out under 40 V in oxalic acid at 7 °C according to the standard procedure [11]: after the first anodization, the disordered porous aluminum oxide layer obtained is removed in chromic acid, then the ordered porous layer is obtained by a second anodization in the same conditions. The length of the pores is defined by the duration of this second anodization. Subsequently (b), the diameter of the pores is increased from its initial value of 40 nm to approximately 60 nm by an isotropic wet chemical etching in warm phosphoric acid. This step maximizes the space available for the electrochemically active material inside the inert matrix. The inner pore walls are coated by ALD (c) by using 3-aminopropyltriethoxysilane, water, and ozone at 150 °C [17–18]. The underlying metallic aluminum substrate is removed oxidatively (d), and the the so-called barrier layer of oxide closing the pore extremities is opened in warm phosphoric acid (e), which leaves a free-standing nanoporous oxide membrane. Its mechanical stability is only sufficient for practical purposes if its thickness is beyond 100 µm. Because of the very large aspect ratio of the pores, the ALD SiO_2_ coating does not reach their lower extremity: in our experimental conditions, the continuous, conformal SiO_2_ coating only reaches a depth on the order of 10 µm. Note that the maximal depth of the deposit could be increased by larger pore diameters and/or optimized experimental conditions [19–20]. The uncoated depth of the aluminium oxide matrix remains chemically and electrochemically inert, and thus functions as the membrane separator that is always placed between both electrodes of batteries. Thus here, the separator and negative electrode are combined into a single unit.

The first five preparative steps (a–e) described above follow literature procedures, whereas the subsequent reduction and the byproduct removal (f,g) are new. In the final step, which again is a standard one, an electrical contact of metallic gold is created on the other side of the membrane by magnetron sputtering (h).

### Investigation of the SiO_2_ reduction on flat samples

**Figure 3:**
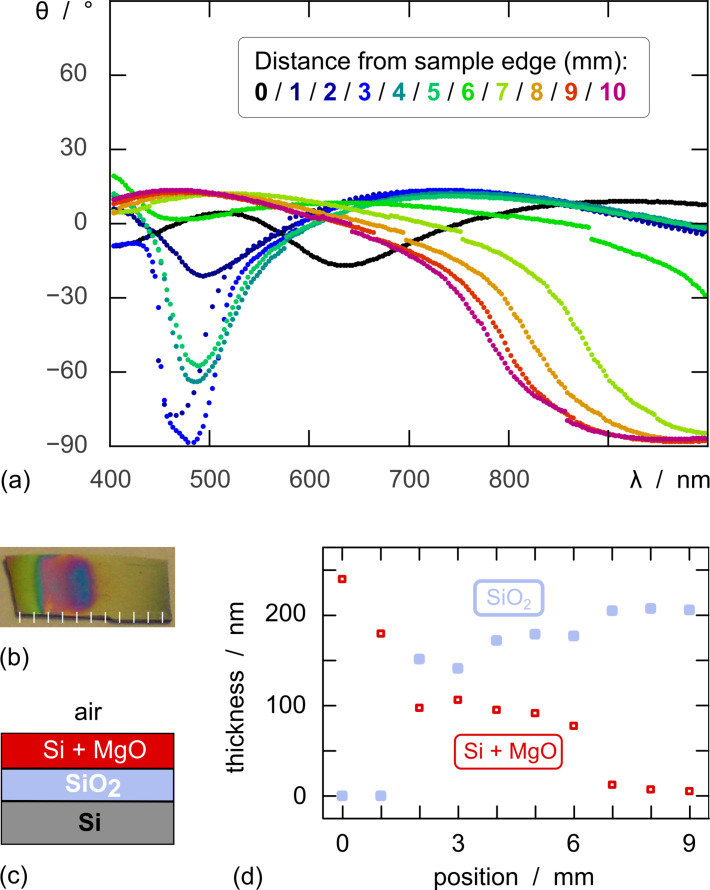
Reaction of a 200-nm thick SiO_2_ layer on a silicon wafer with Mg element at 700 °C: (a) spectroscopic ellipsometry orientation data recorded at various positions of the sample; (b) photograph showing the inhomogeneous reaction extent and the position of the ellipsometric measurements; (c) layer stack used to model the ellipsometry data: the mixed layer is treated as a 1:1 mixture with the Lorentz–Lorentz model; (d) results of the fit: thicknesses of the unreacted SiO_2_ and converted Si + MgO depending on the distance from the sample edge. The experimental data are presented together with the fit curve at each position of the sample in the Supporting Information File 1 (Figure S1).

A native SiO_2_ oxide layer of 200 nm thickness on a crystalline silicon substrate is used as a simple, well-defined model for the initial reactivity tests. We first investigate the reduction with Mg, which has been published [21], and then compare the results with those obtained with Li as the reductant. After having been enclosed in a sealed steel tube in the vicinity of metallic magnesium powder under argon and heated to 700 °C for 7 hours, the wafer piece used as the sample displays a coloration gradient indicative of an incomplete, inhomogeneous reaction (Figure 3b). The result is not improved significantly by longer reaction times or more elevated temperatures. It is consistent with the initial report of this reduction with Mg [21], the authors of which noted that the diatomeous silica used as the substrate turned to a variety of colors from blue (attributed to magnesium silicides) to black (elemental silicon) and brown (incomplete reduction). These qualitative observations can be complemented by quantitative data recorded by spectroscopic ellipsometry. This method analyzes light reflected at the various interfaces present in a thin film sample and enables the experimentalist to determine the layer thicknesses, based on a structural model of the system and the optical properties of the materials involved. In the Si/SiO_2_ sample exposed to Mg vapors, the experimental ellipsometry spectra recorded over the visible wavelength range (Figure 3a) evidence a systematic variation from the part of the sample immediately adjacent to the Mg boat (black and blue curves) to its opposite end (green to pink). This variation can be interpreted based on a model in which an unreacted SiO_2_ film is situated underneath a mixed Si/MgO layer (Figure 3c). If the spectra are fitted to deliver the thicknesses of these two layers (without any constraint on their sum), a clear picture emerges. The reaction extent transitions smoothly from 100% at position 0 to essentially 0% at a distance of 1 cm (Figure 3d and Figure S1 of Supporting Information File 1).

Thus, the reduction by magnesium vapor cannot be exploited on a preparative scale. Among the other metals that can be considered as alternatives to Mg for the reduction of SiO_2_, lithium stands out. Indeed, it is also a strong reductant and provides a negative reaction driving force. Furthermore, lithium also possesses a significant vapor pressure in the range of temperatures considered (Table 1). Its low melting point of 181 °C should be an additional advantage, since it will likely provide faster vaporization kinetics.

**Table 1:** Properties of the metals M = Mg and M = Li of relevance to the thermal reduction of SiO_2_: standard Gibbs free energies of the reactions and metal vapor pressures at two different temperatures [22].

Reaction	Δ_r_*G*°(670 °C) [kJ/mol *e*^–^]	Δ_r_*G*°(700 °C) [kJ/mol *e*^–^]	*p*°(M, 670 °C) [Pa]	*p*°(M, 700 °C) [Pa]

SiO_2_ + 2 Mg ││ Si + 2 MgO	–65	–65	570	1000
SiO_2_ + 4 Li ││ Si + 2 Li_2_O	–56	–56	36	65

**Figure 4:**
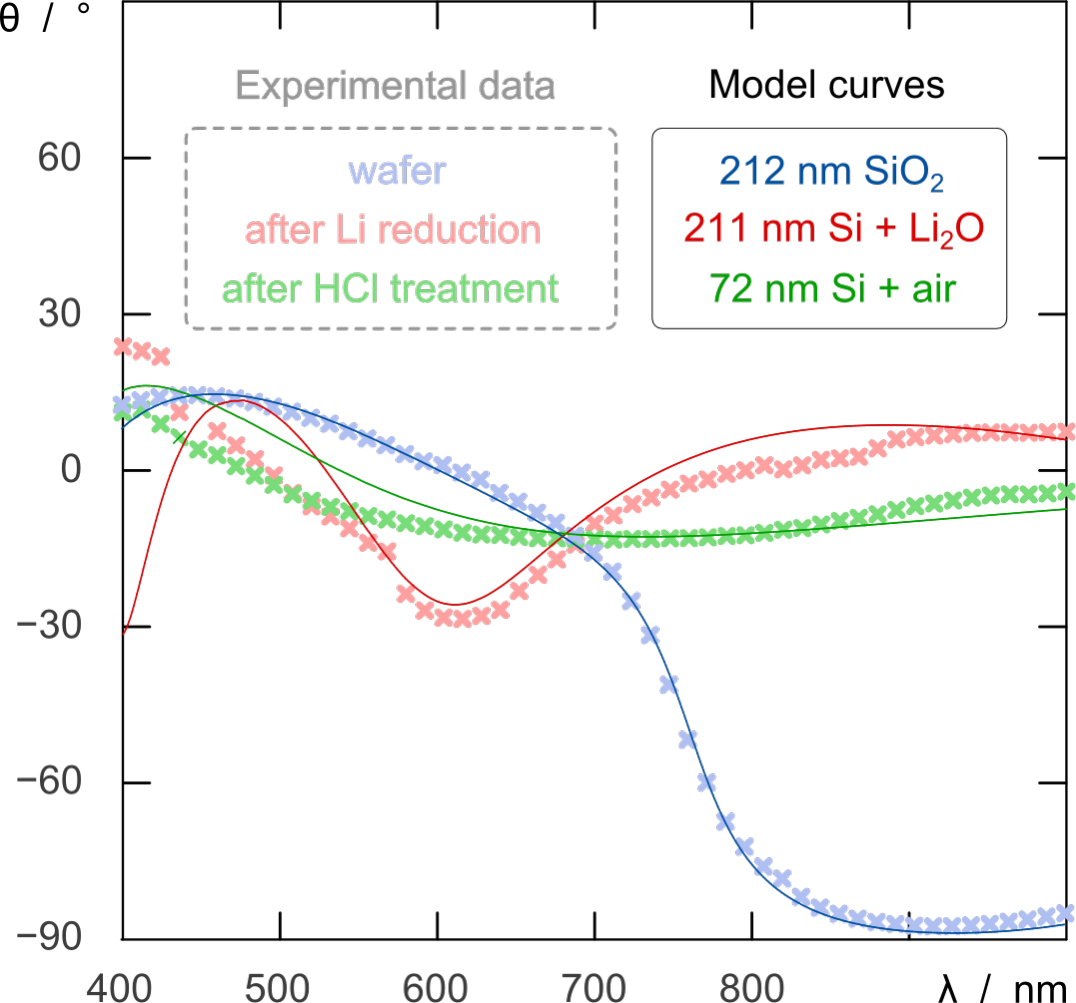
Spectroscopic ellipsometry of flat samples at various stages of preparation: initial substrate with SiO_2_ film (blue), after reaction with Li (red), and after acidic treatment and byproduct removal (green). The experimental data are shown as thick, light crosses, whereas the model curves calculated from the corresponding models are drawn as thin, dark lines.

In fact, we observe that when the reaction of a SiO_2_ film is carried out at 670 °C with lithium instead of magnesium, the reduction is complete, as found by spectroscopic ellipsometry (Figure 4): over the whole sample, the SiO_2_ layer is replaced by what can be modeled as a Si + Li_2_O mixture in volume ratio 2:3. Furthermore, the ellipsometry spectrum changes again upon treatment with a 1 M HCl solution, in a way consistent with a clean conversion to a porous Si layer (modeled as a 5:1 Si/air mixture).

### Application of the thermal SiO_2_ reduction to electrochemically active silicon nanotube arrays

**Figure 5:**
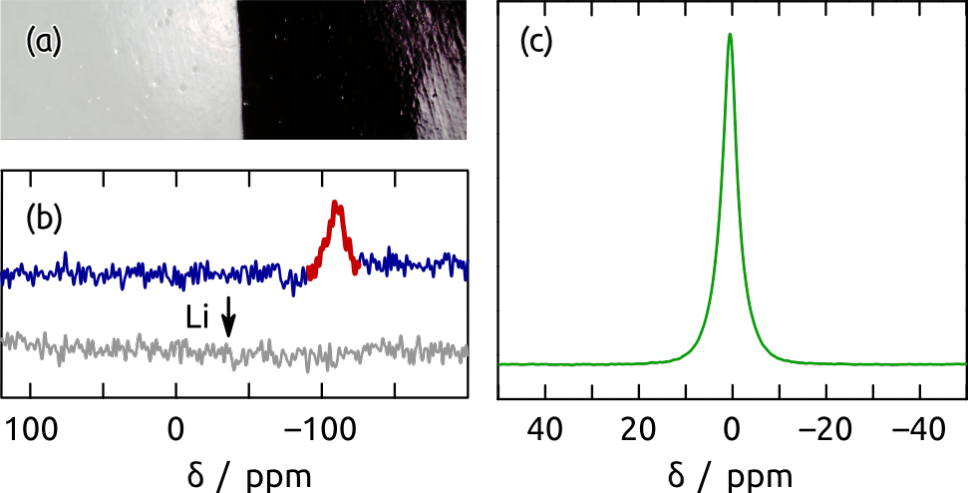
(a) Photograph of two nanoporous samples before and after the reduction by Li vapor with the subsequent acidic treatment (left and right, respectively). (b) ^29^Si MAS NMR spectra before and after reduction. (c) ^7^Li MAS NMR spectrum after reduction.

When a colorless porous sample, obtained as described above (step (e) of Figure 2), is first dried at 400 °C and then submitted to the same reaction conditions including the subsequent HCl treatment (f,g), its appearance turns to a lustrous black (Figure 5a). The conversion can be monitored by magic angle spinning nuclear magnetic resonance (MAS NMR) spectroscopy. In the ^29^Si MAS NMR spectrum of an ALD sample (step (d) of Figure 2), a resonance is observed at −108.4 ppm (Figure 5b), which can be assigned to 'Q' functional groups (Si(OR)_4_, R = Si or C) of siloxane compounds [23]. This is consistent with the identity of the material deposited by the ALD process into the porous samples as SiO_2_. After reduction with Li metal, the ^29^Si NMR signal at −108.4 ppm disappears. This indicates a quantitative conversion of SiO_2_. However, no signal attributable to a crystalline Si phase can be seen. One possible explanation for the absence of ^29^Si NMR signal is the highly amorphous character of Si formed by the reduction reaction. Indeed, extremely broad ^29^Si NMR signals, which are very sensitive to the sample handling conditions, have been reported for Si anode materials [24]. Due to the low natural abundance of ^29^Si and small quantities of the samples available from ALD, the detection of these broad signals can be challenging. Further investigations with ^29^Si-enriched samples are conceivable to examine the reduction product and characterize possible structural changes during electrochemical cycling. In ^7^Li MAS NMR, the reduced material shows a single resonance at +0.5 ppm (Figure 5c), a value characteristic of diamagnetic lithium salts. No signals that could be assigned to metallic lithium and lithium silicide are observed [24]. This substantiates the reaction scheme, in which Li_2_O is produced.

**Figure 6:**
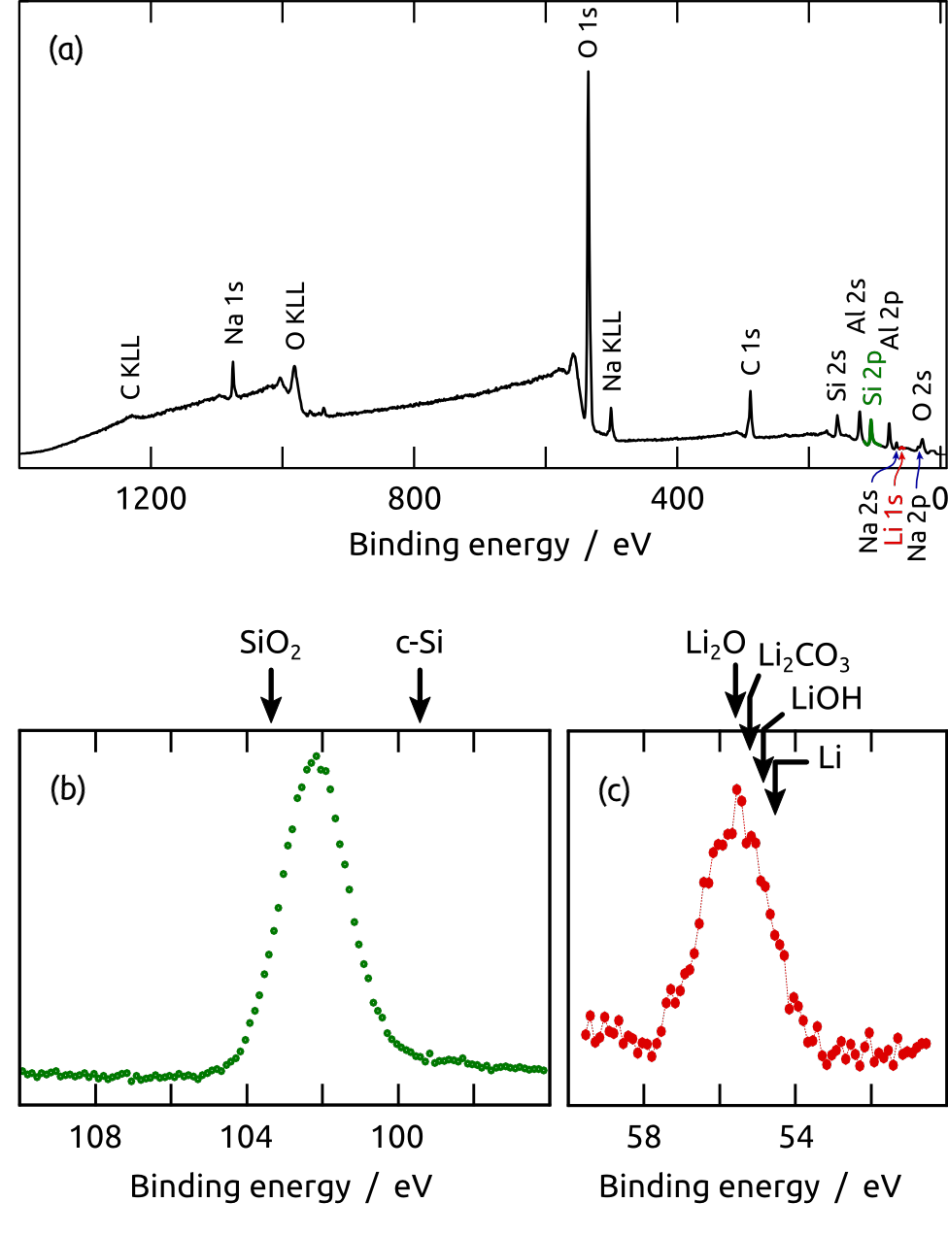
X-ray photoelectron spectrum of a Si nanotube sample at the end of the preparation: (a) survey spectrum, (b) Si 2p peak, and (c) Li 1s peak. The XPS peak positions of reference compounds are given by arrows.

The identity of the final material can be further confirmed by X-ray photoelectron spectroscopy (XPS). The survey XPS spectrum (Figure 6a) shows the signals expected for the elements Al, O, Si, and Li, as well as Na and C contaminants. The Si 2p peak position of 102.2 eV (Figure 6b) unambiguously excludes a significant presence of either crystalline Si (99.3 eV) or SiO_2_ (103.3 eV) [25], in agreement with the ^29^Si NMR data. The peak position is compatible with amorphous silicon, the Si 2p XPS line of which has been found at a somewhat more elevated binding energy than for crystalline silicon [26]. The XPS data do not exclude the possible presence or formation of silane or siloxane species in this system, where Si must be mixed with Li_2_O intimately. The presence of the latter compound is demonstrated by the Li 1s spectrum (Figure 6c), which within the range from 51 eV to 59 eV displays an absolute maximum at 55.6 eV. For reference, the binding energies [27] of metallic Li (54.7 eV), LiOH (54.9 eV), Li_2_CO_3_ (55.2 eV), and Li_2_O (55.6 eV) are indicated in Figure 6. Some contribution of Li_2_CO_3_ (due to aerobic CO_2_ capture) cannot be excluded, since the C 1s spectrum also suggests the presence of carbonates around 290 eV [25].

Scanning electron microscopy (SEM) provides a morphological check of the samples after all preparative steps have been performed. Indeed, the SEM investigation of a sample in cross-section demonstrates that the morphology has been retained throughout the preparative scheme (Figure S2 in Supporting Information File 1).

**Figure 7:**
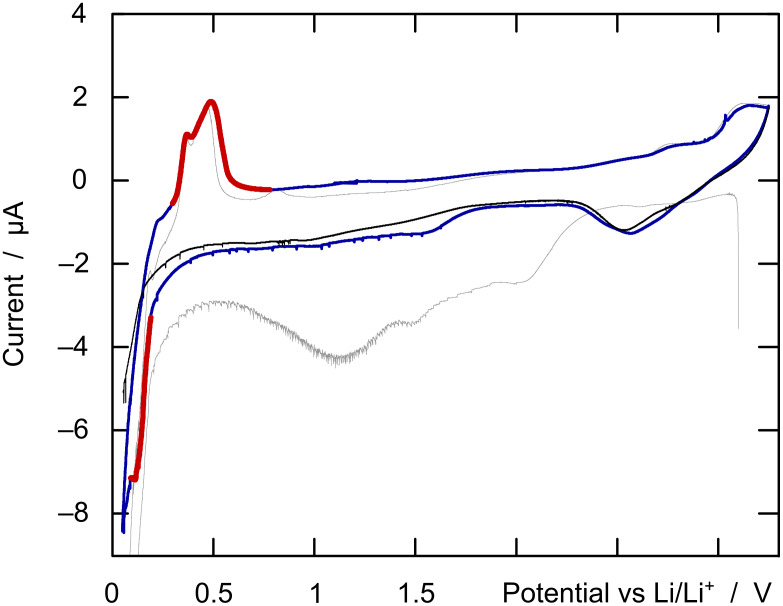
Cyclic voltammetry recorded on a silicon nanotube sample at 0.1 mV s^−1^ in 1 mol L^−1^ LiPF_6_ in ethylene carbonate/dimethylcarbonate by using metallic lithium as the auxiliary electrode and pseudo-reference. The scans were performed between +3.25 V and +0.05 V. The first cycle is plotted as a thin gray line, the second cycle in blue and red, and the third charge in black. The red color highlights the main reduction (charge) and oxidation (discharge) events of the material.

The final demonstration of a successful preparation is provided by the functional test. In the present case, this is the observation of a reduction wave at the expected potential in a Li^+^-containing electrolyte. The cyclic voltammetry of the lithium/silicon system is typically characterized by a sharp reduction between +0.1 and +0.2 V (vs Li/Li^+^) on the charging curve and a broader double oxidation peak situated between +0.3 and +0.7 V upon discharge [6–8]. Upon inclusion as the working electrode into an electrochemical setup with an organic lithium hexafluorophosphate electrolyte and a metallic lithium counter-electrode, our nanotubular silicon samples first display a resting potential on the order of +3.0 V, much more positive than +0.5 V and therefore in line with the chemical identification of the material as elemental silicon above [4]. After the initial charge to +50 mV, slow cyclic voltammetric scans between this value and +3.25 V give rise to the expected curve shape (Figure 7). In fact, the oxidation (discharge) peak appears narrower and at a less positive potential than in most cases reported to date. This could be interpreted as a hint to a particularly good availability of the lithium ions in the solid and their facile extraction out of it, related to the well-defined tubular structure.

## Conclusion

A procedure for the preparation of silicon nanotubes as ordered arrays in an inert matrix has been established. The procedure relies on the combination of a nanoporous 'anodic' template with atomic layer deposition. The lack of an ALD reaction for elemental silicon is circumvented in two steps: the ALD of SiO_2_ is followed by the crucial reaction, a thermal reduction of silicon dioxide to silicon by lithium vapor. The lithium oxide byproduct is removed subsequently. The reduction, performed under argon at 670 °C, is quantitative, homogeneous and well-behaved, in that the product contains neither remnants of silicon oxide nor any lithium silicide, as demonstrated by ellipsometry, MAS-NMR, and XPS. Finally, cyclic voltammetric investigation of the samples testifies to their function as a negative electrode material for lithium ion batteries.

This novel preparative procedure differs from those available to date for making silicon nanotube arrays in three ways [6–8]. Firstly, it is specifically designed so that the experimentalist can tune the geometry of the tubes, that is, their length, diameter and wall thickness, in a systematic and accurate manner. Secondly, the negative electrode is combined with a membrane separator in a single unit. Thirdly, the discharge takes place at a lower potential than in previous comparable systems, a fact that may be indicative of an unusually good availability of the lithium in the electrode material.

## Experimental

### Materials

Metallic lithium granules, magnesium powder, 3-aminopropyltriethoxysilane, oxalic acid, phosphoric acid, copper(II) chloride dihydrate, chromium(VI) oxide, ethanol, hydrochloric acid, perchloric acid, argon, and dioxygen, were purchased from commercial suppliers and used as received. Ozone was generated from dioxygen in a generator BMT 803N from BMT Messtechnik. Aluminum (99.999%) was purchased from Goodfellow. Undoped [100] float-zone silicon wafers with 200 nm thermal oxide were obtained from Si-Mat. Water was purified immediately before use in a Millipore Direct-Q system.

### Instruments

Atomic layer deposition was carried out in a home-built hot-wall reactor equipped with DP-series pneumatic valves from Swagelok and with an MV10C pump from Vacuubrand. Gold was deposited in a Cressington 108 sputter coater. The high-temperature reactions were performed in home-made thick-walled stainless steel cylinders sealed with copper plates, placed in a muffle furnace, model L3/11/P330 from Nabertherm. The reaction cylinders were loaded under argon in an Innovative Technologies InertLab glovebox. The glovebox was equipped with electrical feedthroughs and was also used for the electrochemical measurements. For these, electrochemical potentiostats from Gamry were used (G300 or Reference 600). The electrolyte was 1 M LiPF_6_ in 1:1 ethylene carbonate/dimethylcarbonate (LP71 from Merck) and the counter-electrode was a piece of metallic lithium. The voltammetric curves were recorded at 0.1 mV s^−1^ from the open-circuit potential. Spectroscopic ellipsometry data were collected under a 70° incidence angle with an instrument model EL X-02 P Spec from DRE Dr. Riss Ellipsometerbau GmbH from 400 to 1000 nm. Fits were performed by using the database of material files provided with the instrument. Mixed layers were treated with the Lorentz–Lorentz model as implemented in the software of the instrument. Scanning electron micrographs were taken on a Zeiss Evo equipped with LaB_6_ cathode or a Zeiss Sigma with field emission. Nuclear magnetic resonance spectra were recorded on a Bruker Avance II 400 spectrometer, equipped with a 4-mm magic angle spinning probe. For ^29^Si NMR, 10000 free induction decays were collected at 79.52 MHz with 60° pulses of 3.3 μs and delay times of 20 s. ^7^Li NMR experiments were performed at an operating frequency of 155.56 MHz by using 90° pulses of 3.1 μs and a delay time of 10 s. The ^29^Si and ^7^Li spectra were referenced to tetramethylsilane (TMS) at 0 ppm and to a 1 M LiCl solution (aq) at 0 ppm, respectively. All NMR experiments were carried out at room temperatures and at MAS rates of 10 kHz. XPS spectra were recorded by using a PHI 5600ci multitechnique spectrometer with monochromatic Al K (*hν* = 1486.6 eV) radiation of 0.3 eV full width at half maximum. The resolution of the analyzer is 1.5% of the pass energy, i.e, 0.45 eV. All spectra were obtained by using 400-µm-diameter analysis area. During the measurements, the pressure in the main chamber was kept below 5 × 10^−9^ mbar. Because of the insulating character of the samples, an electrostatic charging effect was observed. All spectra are corrected for this charging effect by using the C 1s line of adsorbed carbon (*E*_B_ = 284.8 eV [23]).

### Preparation steps (a) to (e)

(a) Anodization was performed in home-made two-electrode cells (Figure S3 in Supporting Information File 1) based on a PVC beaker containing the electrolyte. One or several circular openings are at the bottom of the beaker, under which aluminum plates of 2 cm diameter are held between an O-ring and a thick copper plate functioning as the electrical contact. The cell is closed with a lid that holds a silver wire mesh as the counter-electrode and a mechanical stirrer. The copper plate is in contact with a cold plate connected to a closed-circuit cooler by Haake, whereas the setup is thermally insulated laterally. The aluminium plates were first electropolished for 4 min under +20 V in a solution prepared by mixing one part HClO_4_ (70%) with three parts EtOH. They were subsequently rinsed, cooled, and anodized under +40 V for 20 h at 7 °C in 3 M oxalic acid. The oxide was removed by treatment with a chromic acid solution (0.18 M CrO_3_ in 6 wt % H_3_PO_4_) for 20 h at 45 °C. The second anodization was carried out for 60 h in the same conditions as the first anodization. (b) The pores were then widened in 5 wt % H_3_PO_4_ at 45 °C for 10 min. (c) SiO_2_ was deposited by ALD at 150 °C in a three-step reaction based on 3-aminopropyltriethoxysilane (heated to 100 °C), water (maintained at 40 °C) and ozone (delivered at room temperature) [15]. The precursor pulse, exposure and pumping durations were 2/60/90 s, 0.5/60/90 s, and 0.2/60/90 s for the three steps, respectively. (d) The aluminum substrate was subsequently removed by treatment with a 0.7 M CuCl_2_ solution in 10% HCl. The metallic Cu byproduct was removed with concentrated nitric acid. (e) The barrier layer of Al_2_O_3_ closing the pore extremities was opened in 5 wt % H_3_PO_4_ (45 °C, 10 min), after which the samples were dried for 4 h at 400 °C in air.

### Thermal reductions of SiO_2_ and subsequent steps

When flat films (thermal oxide layer on Si wafer pieces) were used as a starting material, their thickness was determined accurately by spectroscopic ellipsometry before reaction. (f) In a glovebox operated under argon, the silicon wafer pieces or nanoporous samples were placed into a stainless steel crucible of approximately 5 × 8 mm^2^ and inserted into a stainless steel cylinder of 10 mm inner diameter, 60 mm length, and 4 mm wall thickness. The magnesium powder or lithium granules were loaded into another crucible and inserted next to the first. The cylinder was closed with two screw nuts sealed with copper plates. A high-temperature copper paste (LiquiMoly 3080) was used to lubricate the screw threads. The sealed cylinder was then heated to the desired reaction temperature (usually 670 or 700 °C) in an oven flushed with nitrogen for several hours (7 h or more), then cooled to room temperature and opened in air. (g) The lithium oxide byproduct was removed by dipping in 1 M aqueous HCl solution for 4 h at room temperature. (h) For electrochemical measurements, a thin gold contact (approximately 50 nm) was finally sputtered onto one side of the sample.

## Supporting Information

The Supporting Information File contains the three Figures S1–S3 mentioned in the text.

File 1 Additional Figures.
