# Supplementary material for: Preparation of electrochemically active silicon nanotubes in highly ordered arrays
Source: Beilstein J Nanotechnol. 2013 Oct 16;4:655–64. doi: 10.3762/bjnano.4.73 (PMC3817651; doi:10.3762/bjnano.4.73)
Supplement: File 1 — Additional Figures. [file Beilstein_J_Nanotechnol-04-655-s001.pdf]

Supporting Information

for

Preparation of electrochemically active silicon nanotubes in highly ordered arrays

Tobias Grünzel[1], Young Joo Lee[1], Karsten Kuepper[2] and Julien Bachmann*[1,3]

Address: [1]Physics Department and Chemistry Department, University of Hamburg, Sedanstrasse 19, 20146 Hamburg, Germany, [2]Physics Department, University of Osnabrück, Barbarastrasse 7, 49076 Osnabrück, Germany and [3]Department of Chemistry and Pharmacy, Friedrich Alexander University Erlangen-Nürnberg, Egerlandstrasse 1, 91058 Erlangen, Germany

Email: Julien Bachmann - julien.bachmann@fau.de

* Corresponding author

Additional Figures

Figure S1: Details of the ellipsometric characterization of a sample from SiO_2 + Mg.

Figure S2: Scanning electron micrograph of a sample at the end of the preparation.

Figure S3: Schematic view of the electrochemical setup used for anodization.

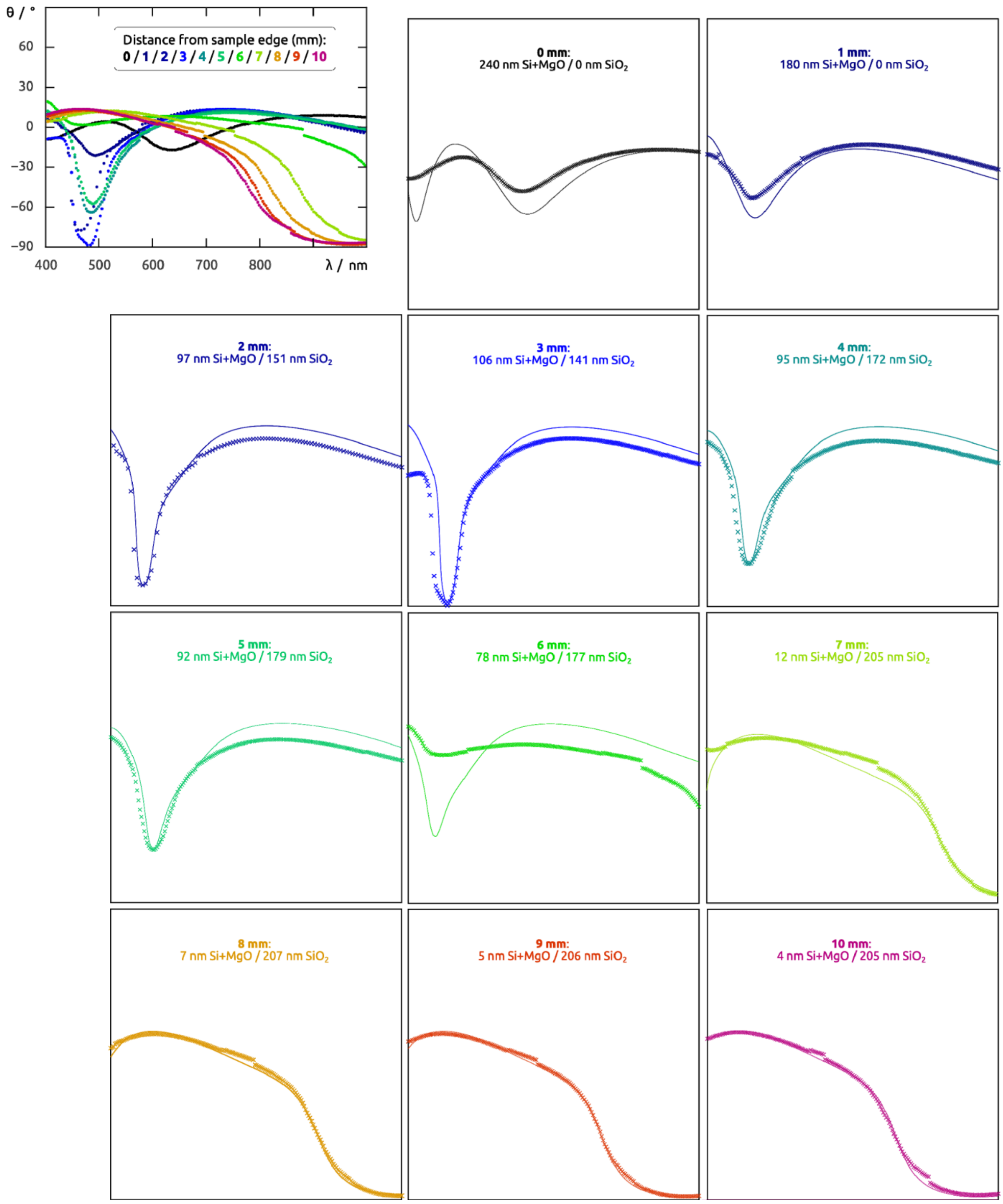

Figure S1: Details of the ellipsometric investigation of the reaction SiO_2 + Mg. The overview presented as Figure 3c of the main text is reproduced in the upper left corner. Every one of the data sets recorded at eleven distinct positions of the sample is reproduced individually in same color on the following panels, together with the calculated curve and the thicknesses corresponding to the calculated curves.

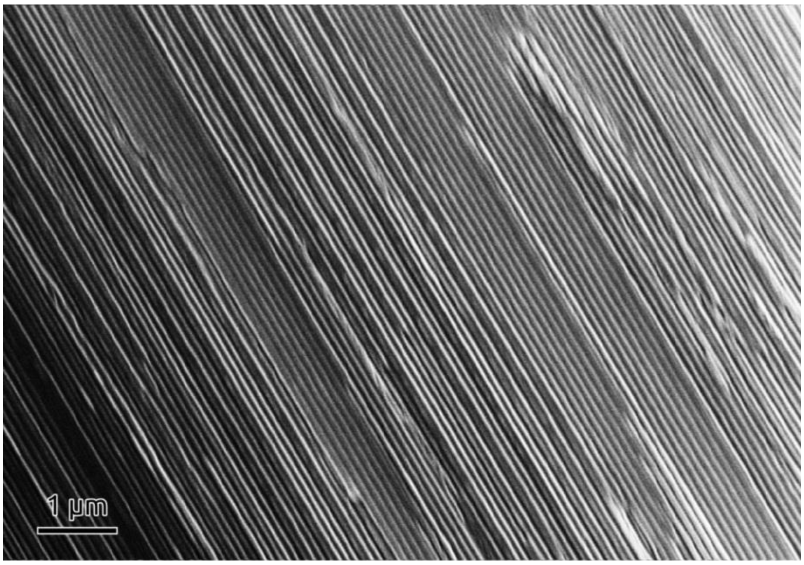

Figure S2: Scanning electron micrograph of a sample at the end of the preparation, presented in cross-section.

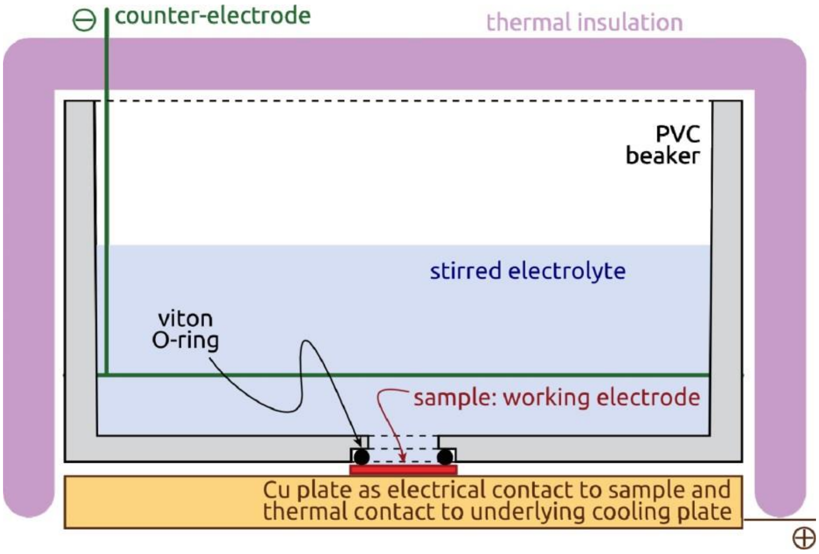

Figure S3: Schematic view of the electrochemical setup used for anodization, step (a) of the procedure described in Figure 2 of the main text.
